# Neoadjuvant Radiochemotherapy Combined with Locoregional Hyperthermia in Locally Advanced Rectal Cancer: Feasibility and Tolerance of Short-Course Versus Long-Course Radiotherapy Schedules

**DOI:** 10.3390/cancers17213529

**Published:** 2025-10-31

**Authors:** Laura Ferrera-Alayón, Bárbara Salas-Salas, Antonio Alayón-Afonso, Miguel Sánchez Carrascal, Laura López Molina, Rafael Alexis Hernández Santana, Hans Crezee, Marta Lloret Sáez-Bravo

**Affiliations:** 1 Department of Radiation Oncology, University Hospital Dr Negrín Las Palmas de Gran Canaria, Barranco de la Ballena s/n, 35010 Las Palmas de Gran Canaria, Spain; aalaafo@gobiernodecanarias.org (A.A.-A.); lolopmol@gobiernodecanarias.org (L.L.M.); rhersanq@gobiernodecanarias.org (R.A.H.S.); mllosae@gobiernodecanarias.org (M.L.S.-B.); 2 Department of Medical Physics, University Hospital Dr Negrín Las Palmas de Gran Canaria, Barranco de la Ballena s/n, 35010 Las Palmas de Gran Canaria, Spain; msancarv@gobiernodecanarias.org; 3 Department of Radiation Oncology, Amsterdam UMC, University of Amsterdam, Cancer Center Amsterdam, 1105 AZ Amsterdam, The Netherlands; h.crezee@amsterdamumc.nl; 4 Department of Clinical Sciences, Faculty of Medicine, Las Palmas de Gran Canaria University (ULPGC), Juan de Quesada, 30, 35001 Las Palmas de Gran Canaria, Spain

**Keywords:** locally advanced rectal cancer, deep hyperthermia, radiative hyperthermia, neoadjuvant chemoradiotherapy, short-course radiotherapy, long-course radiotherapy, treatment tolerance, feasibility study, thermal dose, real-time thermometry

## Abstract

Background: Integrating deep regional hyperthermia (HT) with neoadjuvant chemoradiotherapy (CRT) may enhance treatment efficacy in locally advanced rectal cancer (LARC), yet feasibility and tolerance data remain scarce for both short-course (SCRT) and long-course (LCRT) radiotherapy (RT) regimens. Methods: In this single-center prospective observational study, 67 LARC patients received neoadjuvant RT and chemotherapy (CT) combined with deep radiative HT using a phased-array system (ALBA 4D). Patients treated with SCRT (5 × 5 Gy) were prescribed two HT sessions; those treated with LCRT (25 × 2 Gy) were prescribed ten. HT planning was guided by dedicated software, and real-time thermometry ensured precise thermal delivery. Feasibility was defined as completion of ≥50% of prescribed sessions. Tolerance and toxicity were assessed with standardized clinical scales (QMHT, UMC, CTCAE v4.03). Results: HT was feasible in both groups: 100% of SCRT and 63.6% of LCRT patients completed ≥50% of prescribed sessions. In total, 243 sessions were delivered. Most symptoms were mild and transient, predominantly localized pain. No grade ≥3 HT-related toxicities occurred. All scheduled RT and surgery proceeded without delay. Median T50 was 40.3 °C (SCRT) and 40.4 °C (LCRT); the median RT-to-HT interval was 42 min in both groups. Conclusion: This first Spanish experience shows that deep radiative HT can be seamlessly integrated into both SCRT and LCRT neoadjuvant protocols for rectal cancer. High adherence, favorable tolerance, and reliable thermal control support clinical implementation. Any between-schedule observations are descriptive only; no formal comparative testing was performed. The study was not designed or powered to establish comparative effectiveness between SCRT and LCRT, and the sample size was insufficient to detect rare HT-specific adverse events.

## Simple Summary

Rectal cancer is a common and serious disease that often requires treatment before surgery to improve the chances of removing the tumor completely. This pre-surgery treatment usually includes radiotherapy and chemotherapy. In our hospital, we tested adding a technique called deep hyperthermia, which gently warms the tumor area using a special device. Warming the tumor can make cancer cells more sensitive to other treatments. We applied this approach to 67 patients with rectal cancer before surgery. Some patients received a short, one-week course of radiotherapy, and others received a longer, five-week course. Alongside these treatments, patients had either two or ten heating sessions, depending on the schedule. Most patients completed the planned sessions, and the treatment was generally well tolerated. The most common effects were mild and temporary discomfort, such as local pain, and no serious problems were caused by hyperthermia. All patients received their radiotherapy and surgery on time. These results show that deep hyperthermia can be safely and smoothly combined with standard pre-surgery treatment for rectal cancer. This approach could improve the way this disease is treated, and further research will help to understand its benefits for long-term recovery.

## 1. Introduction

Colorectal cancer is one of the most frequently diagnosed malignancies worldwide, with rectal cancer accounting for approximately one-third of cases [1]. Among these, 5–10% present as locally advanced tumors. The current standard for locally advanced rectal cancer (LARC) is a neoadjuvant approach combining radiotherapy (RT) and chemotherapy (CT) before surgery, which improves resectability, local control and survival [2].Two RT fractionation schedules are commonly used: long-course radiotherapy (LCRT: 50 Gy in 25 fractions with concurrent CT) and short-course radiotherapy (SCRT: 25 Gy in five fractions), as exemplified in the RAPIDO trial [3]. Both regimens are integrated into total neoadjuvant therapy strategies, highlighting the need for optimized preoperative systemic and local treatment.

Deep regional hyperthermia (HT) is a therapeutic technique that elevates tumor temperature to 39–43 °C, enhancing sensitivity to RT and CT through improved oxygenation, inhibition of DNA repair, and immune activation [4,5,6]. Importantly, recent advances have shown that HT also exerts profound immunological effects, including the induction of immunogenic cell death, promotion of dendritic cell maturation, increased T-cell infiltration into the tumor microenvironment, and synergistic activity with radiotherapy and modern immunotherapies [7,8,9]. Malignant tumors, characterized by hypoxia and poor perfusion, are particularly vulnerable to heat [10,11,12]. Clinical studies in several tumor types, including rectal cancer, have demonstrated the efficacy of HT as a radiosensitizer [13,14,15]. Guidelines from the National Comprehensive Cancer Network (NCCN) and the European Society for Hyperthermic Oncology (ESHO) emphasize the importance of quality parameters, such as accurate thermal energy delivery and real-time monitoring, to ensure safe and effective treatments [16,17].

Radiative HT, using phased-array radiofrequency systems, achieves homogeneous heating of deep pelvic tumors [18,19] and offers advantages over capacitive systems [19,20]. Clinical experiences in Europe, including early Spanish programs, have reported its feasibility [18,21]. Moreover, recent studies have shown promising long-term outcomes when HT is added to preoperative chemoradiotherapy (CRT) in LARC [22,23,24]. Importantly, minimizing the interval between RT and HT further enhances radiosensitization [24,25,26].

Based on this background, the aim of the present prospective single-center study was to evaluate the workflow, feasibility, tolerance, and safety of combining deep radiative HT with neoadjuvant CT in patients with LARC treated with either SCRT or LCRT schedules. Any between-schedule findings are presented descriptively, as the study was not powered to test comparative hypotheses.

## 2. Material and Methods

### 2.1. Study Design and Patient Selection

A single-center prospective observational study was conducted between March 2020 and August 2023 at the Radiation Oncology Department of the University Hospital of Gran Canaria Dr. Negrín. All consecutive patients with histologically confirmed, clinically staged II–III rectal adenocarcinoma referred for neoadjuvant chemoradiotherapy during this period were screened through the institutional multidisciplinary tumor board and the radiotherapy scheduling system. Eligible patients were aged 18 years or older with a confirmed diagnosis of LARC and scheduled for neoadjuvant RT with either short-course (SCRT: 5 fractions of 5 Gy) or long-course (LCRT: 25 fractions of 2 Gy) regimens. Concomitant CT was administered according to established protocols. Exclusion criteria included patients under 18 years of age, the presence of metallic prostheses or pacemakers in the treatment area, significant pleural or ascitic effusions, contraindications to treatment such as open wounds, abscesses, or active bleeding, as well as pregnancy or refusal to participate in the study.

The study was approved by the Ethics Committee of the University Hospital of Gran Canaria Dr. Negrín (EUDRACT-2020-335-1). Written informed consent was obtained from all participants prior to initiating treatment.

### 2.2. Radiotherapy and Chemotherapy

RT was delivered using volumetric modulated arc therapy (VMAT) techniques to maximize precision and reduce radiation exposure to adjacent organs at risk (OAR). Treatment plans were created with the ECLIPSE system.

Systemic treatment was administered according to standard-of-care (SOC) protocols tailored to each patient’s clinical condition and compatibility with neoadjuvant RT. In the LCRT group, patients received concurrent capecitabine during RT, with HT delivered immediately after each RT fraction according to schedule. Thus, HT was fully integrated into the concurrent chemoradiotherapy regimen. In the SCRT group, patients were treated following a total neoadjuvant therapy (TNT) approach (as in the RAPIDO trial [3]), where HT was delivered after RT fractions, and systemic chemotherapy was administered sequentially after completion of SCRT.

### 2.3. Deep Hyperthermia

Deep locoregional HT was administered using the ALBA 4D system (ALBA 4D system (Medlogix Srl, Rome, Italy) This system employs a multi-beam phased array of four waveguide antennas operating at 70 MHz, which independently modulate beams in phase and amplitude. The phase and amplitude control of this configuration allows precise collimation of energy to the rectal tumor site at the required target location, optimizing therapeutic outcomes while minimizing unintended excessive heating of surrounding tissues resulting in treatment-limiting hot spots [20].

#### 2.3.1. Planning and Delivery

Treatment planning was conducted in two steps. Easy Plan (Medlogix Srl, Rome, Italy) created the geometrical HT plan, ensuring precise spatial alignment of the treatment field with the patient’s anatomy and tumor geometry. Subsequently, Plan 2 Heat (Medlogix Srl, Rome, Italy) was used to optimize dosimetry by incorporating patient-specific tissue density, ensuring compatibility with RT plans and allowing for the identification and mitigation of potential hotspots.

During HT patients were positioned supine on a mattress, with the target region lying on a water bolus and with a water bolus between patient and the antennas to enhance energy delivery and ensure proper contact between the applicator and the body surface. HT sessions started with a 10–30 min warming-up time until therapeutic temperatures of ≥40 °C were reached, after which the steady state phase started which lasted 60 min, sessions were scheduled twice weekly (every 72 h). Patients undergoing LCRT were prescribed 10 HT sessions twice a week, patients receiving SCRT were prescribed 2 HT sessions.

#### 2.3.2. Monitoring and Control

Thermometry is used [16] to ensure effective monitoring and precise energy delivery. A total of 64 temperature sensors were used, strategically placed near the tumor site to collect real-time data on temperature distributions. Rectal pelottes of standard dimensions were utilized for temperature monitoring. A water bolus, available in sizes S, M, and L, was used to facilitate effective skin cooling and to focus the energy precisely on the tumor site.

#### 2.3.3. CT Simulation

CT simulation was performed to support both RT and HT planning. For RT, patients were positioned prone to facilitate optimal dose distribution and minimize organ motion. In contrast, for hyperthermia, patients were positioned supine, aligned with the same water bolus used during treatment.

To improve reproducibility, longitudinal tattoos and lateral laser alignment were employed, ensuring consistent patient setup across all sessions. Additionally, a towel was placed beneath the water bolus to clearly differentiate the bolus from the patient’s body in imaging. A radiopaque marker was positioned in the anus to assist in precise localization of the rectum. The CT protocol included imaging with 30 cm margins above and below the isocenter to comprehensively capture the treatment area and adjacent anatomy.

#### 2.3.4. In Vivo Thermometry

Real-time minimally invasive thermometry was integral to hyperthermia sessions. Temperature monitoring was achieved using up to 64 sensors strategically placed in proximity to the tumor in the rectum, bladder and vagina. Sensors were categorized based on their location: T1 was positioned within the tumor, while T2–T4 were placed in peripheral regions, and N sensors served as distant reference points. Data from these sensors enabled immediate identification of hotspots, allowing for real-time adjustments to energy delivery and ensuring optimal thermal distribution throughout the session, in accordance with quality assurance recommendations for deep hyperthermia [27].

#### 2.3.5. Integration HT with RT Patient Systems

The integration of HT with RT was facilitated using the ECLIPSE platform for RT planning, while HT planning platforms Easy Plan and Plan 2 Heat interfaced seamlessly to ensure alignment between hyperthermia and radiotherapy treatment plans. The ARIA Oncology Information System (OIS) managed DICOM worklists, enabling efficient communication between planning and delivery systems. The ALBA 4D system was thus fully integrated into the hospital network.

#### 2.3.6. Workflow

The workflow for integrating HT with RT was designed to ensure efficiency and patient-centered care. On the same day, patients underwent a comprehensive evaluation by the physician and nursing team, followed by CT simulation for both RT and HT planning. The treatment planning phase involved the creation of geometrical and dosimetric plans for HT using Easy Plan and Plan 2 Heat, which were synchronized with RT plans developed in ECLIPSE.

Scheduling of RT and HT sessions was coordinated to optimize timing and minimize patient burden. A nurse was present during all HT sessions to monitor patient safety and comfort. Weekly evaluations by the physician were conducted to assess tolerability, toxicity, and overall progress. Post-treatment follow-up was conducted regularly to evaluate outcomes and ensure long-term monitoring.

### 2.4. Adherence, Tolerance and Toxicity

Adherence to treatment is a critical feasibility parameter and was defined as the successful completion of a sufficient number of the prescribed hyperthermia sessions. Specifically, we considered the adherence criterion met when patients completed more than 50% of the prescribed sessions, in line with definitions used in previous HT feasibility trials [17,21]. Because HT was administered every 72 h and only during RT, the total number of prescribed sessions differed between schedules: two sessions for SCRT and up to ten sessions for LCRT. Notably, previous studies have reported that treatment adherence may become more challenging over time, particularly when acute RT-associated toxicity emerges during LCRT regimens [28]. For instance, the German experience in rectal cancer with regional HT showed that patient tolerance decreased after week 3, prompting protocol adjustments to deliver HT twice per week during the initial weeks and reducing to once weekly thereafter to maintain adherence and minimize drop-outs [28].

Tolerance and toxicity were monitored throughout the study. The tolerability of HT sessions was assessed using the Quality Management in Hyperthermia (QMHT) scale, with patient-reported outcomes collected via the UMC scale. Pain intensity was specifically assessed using the Numeric Rating Scale (NRS, 0–10). Treatment-related adverse events were graded according to the Common Terminology Criteria for Adverse Events (CTCAE v4.03) [29]. These evaluations ensured a comprehensive understanding of patient responses and facilitated real-time adaptations when necessary. In some cases, interruptions of HT sessions were required due to patient-reported discomfort such as pain, thermal intolerance, or anxiety. While efforts were made to resume treatment during the same session, in certain instances the session had to be discontinued or cancelled. In addition to intolerance specifically related to HT, interruptions or cancellations could also result from emerging toxicities associated with concomitant RT or CT (e.g., proctitis, fatigue, or gastrointestinal symptoms). Patients experiencing significant discomfort or treatment-related toxicity were evaluated by the physician on the same day, and when appropriate, supportive measures—including analgesics, anxiolytics, anti-inflammatory drugs, or specific medical interventions—were prescribed to improve tolerance and facilitate the continuation of treatment in subsequent sessions.

### 2.5. Statistical Analysis

All analyses were descriptive. Continuous variables are reported as means, medians, and ranges and categorical variables as frequencies and percentages. No formal hypothesis testing between the short-course and long-course schedules was planned. The study was not designed or powered to establish comparative effectiveness or to detect rare hyperthermia-specific adverse events. Analyses were performed using IBM SPSS Statistics for Mac, version 26.

## 3. Results

### 3.1. Patient Characteristics

Between March 2020 and August 2023, a total of 67 patients (34 in the SCRT group and 33 in the LCRT group) with LARC were enrolled in the study. The mean age was 64.5 years (range: 36–80 years), with the majority being male (44 out of 67; 65.7%). Most patients (60 out of 67; 89.6%) presented with stage III LARC. Detailed patient demographics, tumor characteristics, and associated treatments are summarized in Table 1.

### 3.2. Feasibility

HT was feasible in most patients. All 34 SCRT patients (100%) completed at least one session (range: 1–2), and 31 out of 34 SCRT patients (91.18%) completed both prescribed sessions. In the LCRT group, 21 out of 33 patients (63.64%) completed at least five sessions (range: 1–10). Treatment interruptions were observed in 23 patients (34%), primarily due to pain in the treatment area (47.8%), RT-related toxicity (21.7%), technical issues (17.4%), pressure from the water bolus (8.7%), and claustrophobia (4.3%). Importantly, all patients completed their prescribed RT regimens without interruptions and underwent surgery within the planned timeframe, highlighting the feasibility of integrating HT into neoadjuvant treatment protocols for LARC.

Over this period, 243 HT sessions were conducted, 65 sessions for 34 patients undergoing SCRT and 178 sessions for 33 patients receiving LCRT. Patients in the SCRT group typically received 1–2 sessions per patient, with an average of 1.91 sessions per patient. Patients in the LCRT group received an average of 5.12 sessions, with a broad range of 1–10 sessions per patient. These differences are descriptive, as no formal statistical comparisons were performed.

### 3.3. Tolerance

Most patients reported manageable symptoms during HT, predominantly localized pain in the gluteus, abdomen, pelvis, and thighs. Other reported sensations included skin warmth, discomfort, and pressure from the water bolus. A detailed breakdown of the observed effects is presented in Table 2.

### 3.4. Toxicity

There were no treatment interruptions due to toxicity that affected the completion of RT or the planned surgical timeline. Furthermore, no HT-specific adverse events, such as severe burns or heat intolerance, were observed, indicating that the addition of HT did not contribute to treatment-related toxicity (Table 3).

A total of 15 grade 3 events of toxicity were recorded, all attributed to RT and CT. These included rectal mucositis in 4 patients (6%), radiodermatitis in 4 patients (6%), hemorrhoids in 4 patients (6%), diarrhea in 2 patients (3%), and proctitis in 1 patient (1.5%).

All other toxicities were grade 1 or 2, with no grade 4 toxicities reported.

Additionally, an exploratory analysis using data from our institutional registry—including patients treated before, during, and after the implementation of hyperthermia—revealed no significant differences in the incidence or severity of acute toxicities between those treated with and without hyperthermia (Supplementary Table S1). It should be noted that hyperthermia itself does not add systemic or gastrointestinal toxicity to chemoradiotherapy; its impact is limited to session-related tolerance (e.g., local sensations of heat or pressure), which were mild and transient. This supports the safety of integrating deep regional hyperthermia into standard neoadjuvant treatment.

### 3.5. Thermal Dose Coverage/HT Parameters

Thermal dose values were recorded for both treatment groups, including median and range values for T50. In the SCRT group, the median T50 was 40.33 °C, ranging from 38.72 °C to 41.45 °C, while in the LCRT group, the median T50 was 40.44 °C (range: 38.56 °C to 41.66 °C). The complete set of thermometric parameters, including T10, T90, Tmin, Tmax, and Tavg, are detailed in Table 4.

The time between the end of RT (beam-off) and the start of HT was similar in both groups, with a median of 42 min in the SCRT group and 41 min in the LCRT group (Table 5).

The median duration of the HT session was 60 min in both groups, with mean durations of 54 min (SCRT) and 50.9 min (LCRT) (Table 5).

## 4. Discussion

The combination of RT with HT has demonstrated clinical evidence of synergistic effects in various tumor types, including rectal cancer [4,5,14,15,22,26]. HT improves tumor perfusion, increases oxygenation, and inhibits DNA repair, leading to enhanced radiosensitization and chemotherapy efficacy [4,5]. In addition to these radiosensitizing mechanisms, HT also acts as an immune modulator by inducing heat shock protein release, promoting antigen presentation, and stimulating both innate and adaptive immunity [7,8]. These effects contribute to better local control and may optimize treatment response in neoadjuvant settings. However, achieving these benefits requires strict adherence to hyperthermia quality parameters, as recommended by the European Society for Hyperthermic Oncology (ESHO) [16,25,28]. These parameters include precise temperature monitoring, standardized treatment planning, and real-time energy control to ensure uniform heat distribution and optimal dosing [18]. Our study adhered to these standards, integrating HT with CRT in LARC and demonstrating its feasibility in routine clinical practice. Recent reviews also emphasize the role of HT in inducing immunogenic cell death and fostering a tumor microenvironment permissive to immune cell infiltration [7].

### 4.1. Feasibility and Adherence to Protocol

At our center, we have successfully integrated RT with HT treatment, ensuring a smooth integral workflow. After completing the RT session, the patient proceeds directly to the HT treatment room, where the session can start without delay.

One of the key findings of this study was the high adherence rate to HT sessions. Among the 67 patients included, all 34 SCRT patients (100%) completed at least one session, accounting for 50% of the prescribed treatment. Additionally, 31 out of 34 SCRT patients (91.18%) successfully completed both prescribed sessions, demonstrating a high adherence rate. In our cohort, adherence appeared higher with the SCRT schedule; however, these observations are descriptive and causality cannot be inferred from this observational design.

This excellent adherence in the SCRT group may be related to the fact that most of the acute side effects of CRT (such as gastrointestinal toxicity or fatigue) do not yet manifest during the short 5-day treatment period. Therefore, tolerance remains high, and no significant RT-related toxicity interferes with the completion of HT.

Adherence in the LCRT group showed a different pattern. 21 out of 33 LCRT patients (63.64%) completed at least five sessions, with a mean of 5.12 completed sessions, a median of 5 sessions and the number of sessions ranging from 1 to 10. Notably, this median is higher than that reported previously [30], where the number of HT sessions ranged from 1 to 9, with a median of 4 sessions. The median number of completed sessions in our cohort was higher than in some previous reports (e.g., Gani et al.), which may reflect differences in workflow, patient selection, or measurement. These observations are descriptive, and no formal statistical comparisons were performed. It is worth noting that, in the German experience reported previously [22], the treatment protocol anticipated the decline in patient tolerance over time and therefore started with two HT sessions per week during the initial phase of LCRT, aiming at completing approximately six sessions in total. This proactive approach was designed to complete the majority of HT treatments before cumulative toxicity compromised adherence.

These results confirm the feasibility of integrating HT into both SCRT and LCRT ra schedules in a real-world setting. Previous studies combining HT with RT have reported similar feasibility outcomes, although adherence rates can vary depending on treatment tolerability and patient-specific factors [25,26]. Notably, our cohort exhibited a treatment interruption rate of 34%, which was lower than in previously reported studies, where discomfort and logistical challenges often impacted treatment completion [20]. It should be noted that the latter studies used different HT equipment, such as capacitive systems, which offer less control over energy distribution and present greater difficulty in effectively heating deep pelvic tumors like rectal cancer [19,20]. This difference may be partly explained by the variation in equipment and treatment delivery approaches. Importantly, in our protocol HT was consistently delivered after RT on the same day, with a median RT–HT interval of approximately 41–42 min. This sequence was chosen based on prior evidence showing that for RT followed by HT shorter intervals maximize the radiosensitizing effect of HT [24]. We did not evaluate pre-RT HT, and therefore no direct conclusions regarding that approach. Exploratory descriptive analyses indicated that per-session thermal parameters (e.g., median T50 and RT–HT interval) were highly comparable between SCRT and LCRT schedules, suggesting similar radiosensitizing conditions. The main difference lays in cumulative exposure: while all SCRT patients completed the two prescribed sessions, adherence decreased across the longer LCRT regimen. These findings are consistent with previous feasibility studies in rectal cancer [21,23].

### 4.2. Hyperthermia Sessions Administered and Treatment Tolerance

A total of 243 HT sessions were conducted, with patients in the SCRT group receiving between 1 and 2 sessions per patient (mean: 1.91) and those in the LCRT group receiving between 1 and 10 sessions per patient (mean: 5.12, median: 5).

One of the key advantages of real-time thermometric control during sessions is the ability to dynamically adjust treatment parameters, such as antenna power and other technical settings, to enhance patient tolerance and optimize heat distribution. This level of control allows for individualized adjustments that improve tumor temperature, overall comfort, and adherence to treatment.

Treatment interruptions occurred in 23 patients (34%), with the primary causes pain in the treatment area (47.8%), radiotherapy-related toxicity (21.7%), and technical issues (17.4%). However, despite these interruptions, all patients successfully completed their prescribed RT regimens and underwent surgery within the planned timeframe, confirming the feasibility of integrating HT without compromising standard oncological treatment protocols.

Our findings align with data from other deep HT programs in previous studies, where treatment adherence and feasibility were influenced by technological advancements in treatment planning and real-time temperature monitoring [21]. The improved adherence observed in our cohort could be attributed to an optimized workflow, increased clinician experience, and the consistent use of HT treatment planning guidance (EasyPlan and Plan 2 Heat), which ensured individualized optimized thermal dose delivery by allowing precise modulation of treatment parameters based on continuous thermometric feedback and online adaptive HT planning guidance [18].

### 4.3. Toxicity Profile of the Combined Treatment

No grade 3 or higher toxicities directly attributable to HT were observed, and no patients experienced severe burns or heat intolerance. The recorded grade 3 toxicities, including mucositis, radiodermatitis, and proctitis, were attributable to CRT and are consistent with the expected toxicity profile of neoadjuvant treatment protocols. These findings align with previous studies demonstrating that HT does not significantly increase overall treatment-related toxicity when applied under standardized conditions [17,23].

Additionally, most treatment-related symptoms were mild and transient, and could be resolved by changing system settings, with the most common being localized pain in the gluteus (59.7%), abdomen (49.2%), and pelvis (43.3%). In the series by Hamazoe et al. (1991), 27% of patients treated with capacitive HT reported discomfort that affected treatment adherence [31]. Although a higher proportion of patients in our study reported some form of excess heating sensation during sessions, radiative HT allowed for a more homogeneous heat distribution, strong skin cooling avoiding energy accumulation in superficial tissues and reducing dermal overheating, which is a major limitation of capacitive HT [19].

### 4.4. Workflow Optimization and Beam-Off Times

A major benefit of our study design was the administration of HT following radiotherapy, which reduced waiting times and improved overall treatment efficiency. The time interval between the end of RT (beam-off) and the start of HT has been recognized as a key parameter of treatment efficacy and quality in RT-HT combination protocols.

In our study, the mean beam-off to start HT steady state interval was 42 min, with comparable medians across schedules; no formal statistical testing was performed. The median beam-off to start HT steady state interval was 42 min in the SCRT group and 41 min in the LCRT group, which is consistent with previous studies emphasizing the importance of minimizing this interval to maximize hyperthermia’s radiosensitizing effect [24,26]. These workflow metrics are based on a single-center observational experience and should be interpreted descriptively.

Maintaining a short and standardized beam-off time is crucial, as it directly impacts the effectiveness of HT in enhancing radiation-induced tumor cell damage. Our results suggest that the integration of optimized workflow protocols allowed for a fast and efficient transition from RT to HT, ensuring conditions favorable for optimal thermal enhancement of RT effects.

### 4.5. Thermal Dose Coverage and HT Parameters

Temperature data analysis revealed that thermal dose coverage remained within therapeutic ranges across both treatment groups, demonstrating a homogeneous treatment application. The median T50 was 40.33 °C in the SCRT group and 40.44 °C in the LCRT group, with a range of 38.72 °C to 41.45 °C in SCRT and 38.56 °C to 41.66 °C in LCRT. These values align with previously established temperature thresholds for effective HT treatment [16,27]. The similarity in T50 and T-average values between the two treatment groups further supports the homogeneity of HT delivery across the target areas.

The ability to achieve and maintain uniform thermal dose distribution throughout treatment sessions was made possible by a comprehensive thermometry approach using 64 sensors in the target region, which allowed for real-time monitoring of temperature parameters. This extensive thermometric control ensured precise temperature regulation, minimized thermal variability, and reduced the risk of localized overheating. Thermometry precision was further enhanced by using and visualizing continuous real-time data collection, enabling rapid adjustment of treatment parameters when needed, thus ensuring optimal therapeutic heating throughout the session.

Differences between radiative and capacitive HT systems have been widely studied, particularly in simulation-based analyses evaluating their ability to achieve deep-seated tumor heating while maintaining safety in surrounding tissues. Kok et al. conducted a simulation study comparing both methods, demonstrating that radiative hyperthermia provides superior loco-regional heating due to its deeper penetration and good target focusing, resulting in improved thermal dose homogeneity [19]. These findings reinforce the benefits of radiative HT in ensuring consistent thermal coverage across treatment areas while avoiding the overheating of superficial tissues, a common limitation of capacitive HT systems.

The importance of real-time thermometry in HT treatment planning has been well-documented in the literature, reinforcing its role in optimizing treatment delivery, ensuring patient safety, and improving treatment reproducibility [28]. The combination of realizing consistent T50 values, real-time thermometry, and use of treatment planning ensured that HT treatments in our study were administered homogeneously and effectively, maintaining stable and optimal temperature levels throughout the sessions.

### 4.6. Strengths and Limitations

This study represents the first experience in Spain integrating radiative HT with neoadjuvant CRT for LARC. One of its main strengths is the demonstration—within a prospective observational design—of the feasibility of incorporating deep HT into different multimodal treatment protocols while maintaining adherence to oncological standards. The successful integration of HT with a short-course radiotherapy schedule of just one week is completely novel.

A key contribution of this study is the establishment of an optimized workflow that complies with quality parameters for HT, particularly the beam-off to start HT steady state time. Maintaining a consistent and controlled beam-off interval plays a critical role in optimizing the radiosensitizing effects of HT, and our results show that this interval can be standardized in routine clinical practice. Despite our limited prior experience with this technique—introduced in our center in 2020—we have successfully implemented a structured workflow aligned with the standards set by the ESHO. This suggests that deep HT can be integrated even in centers with developing expertise, provided that strict treatment protocols and quality control measures are followed.

Moreover, the use of advanced planning systems allowed for precise and individualized energy delivery, which may have contributed to high adherence rates and lower levels of discomfort in our cohort. The ability to adjust treatment parameters in real time, based on comprehensive thermometric monitoring, further ensured a homogeneous and effective HT application.

However, several limitations must be acknowledged. The single-center nature of the study limits generalizability, and the sample size—while adequate for descriptive conclusions on feasibility and tolerance—was not designed or powered to detect rare HT-specific adverse events or to establish comparative effectiveness between SCRT and LCRT schedules. All between-schedule observations are descriptive, as no formal statistical comparisons were performed. The absence of randomization or a direct comparison with capacitive HT also limits our ability to attribute the observed differences exclusively to the radiative technique. Furthermore, the lack of long-term oncological follow-up precludes conclusions on disease control or survival outcomes.

Despite these limitations, our findings provide valuable real-world evidence supporting the feasibility of integrating deep regional HT into standard neoadjuvant treatment protocols. Moreover, growing evidence highlights the synergistic potential of HT not only with radiotherapy and chemotherapy but also with immunotherapy, providing a strong translational rationale for combined treatment strategies [9,32]. Future studies with larger sample sizes, longer follow-up periods, and comparative analyses will be essential to further define the role of radiative HT in the management of locally advanced rectal cancer.

## 5. Conclusions

This prospective single-center observational study demonstrates that deep radiative HT can be feasibly and safely integrated into both SCRT and LCRT neoadjuvant CRT protocols for LARC. High adherence rates, favorable tolerance, and consistent achievement of thermal targets were observed, without delaying planned RT or surgery.

All between-schedule findings are descriptive, as no formal statistical comparisons were performed, and the study was not designed or powered to establish comparative effectiveness or detect rare HT-specific adverse events. These results support the technical and logistical feasibility of incorporating radiative HT into routine clinical workflows, while underscoring the need for larger, multicenter, randomized trials with long-term follow-up to assess oncological outcomes and define its role in standard treatment algorithms.

## Figures and Tables

**Table 1:** Patient Demographics, Tumor Characteristics, and Treatment Details. This table summarizes the patient characteristics, including sex, tumor location, tumor stage, RT schedule, and CT information. Data are presented as the number of patients (n) and percentage (%).

	n/(%)
**Sex**	
Male	23 (34.3%)
Female	44 (65.7%)
**Tumor Location**	
High	12 (17.9%)
Medium	40 (59.7%)
Low	15 (22.4%)
**Tumor Stage**	
II	7 (10.5%)
III	60 (89.6%)
RT schedule	
SCRT	34 (50.7%)
LCRT	33 (49.3%
**CT**	
No	1 (1.5%)
Yes	66 (98.5%)
**Type of CT**	
Folfox	15 (22.4%)
Capox	16 (23.9%)
Capecitabine	35 (52.2%)

**Table 2:** Effects observed by patients during HT sessions. Some patients encountered multiple effects throughout the sessions.

Effects on Patients	Number of Patients (n)	Percentage (%)
**Pain (NRS)**		
Gluteus	40	59.7
Abdomen	33	49.2
Pelvis	29	43.3
Thighs	27	40.3
Dorsolumbar region	10	14.9
Lower limbs	5	7.5
**Sensations**		
Warm skin	21	31.3
Discomfort	15	22.4
Pressure from the bolus	14	20.9
Tingling	8	11.9
Deep pressure	7	10.4
Arterial hypotension	4	6
**Other Effects**		
Claustrophobia	3	4.5
Urinary urgency	2	3
Bradycardia, Tachycardia, Arterial hypertension, Dizziness, Shortness of breath	1	1.5

**Table 3:** Summary of toxicities. Table summarizing the incidence and severity of toxicities observed in the cohort treated with RT, CT, and HT. Data are presented as the number of patients (n) and percentage (%). Toxicity was evaluated using CTCAE v4.03.

Toxicity CTCAE v4.03	No Toxicity (Grade 0)	Mild–Moderate (Grade 1–2)	Severe (Grade 3)	Very Severe (Grade 4)
**Anal pain**	55 (82.09%)	12 (17.91%)	0 (0.0%)	0 (0.0%)
**Anal ulcer**	67 (100.0%)	0 (0.0%)	0 (0.0%)	0 (0.0%)
**Constipation**	52 (77.61%)	15 (22.39%)	0 (0.0%)	0 (0.0%)
**Diarrhea**	42 (62.69%)	23 (34.33%)	2 (2.99%)	0 (0.0%)
**Fecal incontinence**	59 (88.06%)	8 (11.94%)	0 (0.0%)	0 (0.0%)
**Flatulence**	63 (94.03%)	4 (5.97%)	0 (0.0%)	0 (0.0%)
**Hemorrhoids**	46 (68.66%)	17 (25.37%)	4 (5.97%)	0 (0.0%)
**nausea**	57 (85.07%)	10 (14.93%)	0 (0.0%)	0 (0.0%)
**proctitis**	51 (76.12%)	15 (22.39%)	1 (1.49%)	0 (0.0%)
**Rectal fissure**	67 (100.0%)	0 (0.0%)	0 (0.0%)	0 (0.0%)
**Rectal fistula**	67 (100.0%)	0 (0.0%)	0 (0.0%)	0 (0.0%)
**Rectal bleeding**	64 (95.52%)	3 (4.48%)	0 (0.0%)	0 (0.0%)
**Rectal mucositis**	48 (71.64%)	16 (23.88%)	3 (4.48%)	0 (0.0%)
**Rectal obstruction**	67 (100.0%)	0 (0.0%)	0 (0.0%)	0 (0.0%)
**Rectal pain**	39 (58.21%)	28 (41.79%)	0 (0.0%)	0 (0.0%)
**Vomiting**	65 (97.01%)	1 (1.49%)	1 (1.49%)	0 (0.0%)
**Radiodermatitis**	34 (50.75%)	29 (43.28%)	4 (5.97%)	0 (0.0%)
**Myalgia**	66 (98.51%)	1 (1.49%)	0 (0.0%)	0 (0.0%)
**Anxiety**	63 (94.03%)	4 (5.97%)	0 (0.0%)	0 (0.0%)
**Dysuria**	57 (85.07%)	10 (14.93%)	0 (0.0%)	0 (0.0%)
**Hematuria**	66 (98.51%)	1 (1.49%)	0 (0.0%)	0 (0.0%)
**Urinary frequency**	67 (100.0%)	0 (0.0%)	0 (0.0%)	0 (0.0%)
**Urinary incontinence**	67 (100%)	0 (0.0%)	0 (0.0%)	0 (0.0%)
**Urinary tract pain**	66 (98.51%)	1 (1.49%)	0 (0.0%)	0 (0.0%)
**Urinary urgency**	61 (91.04%)	6 (8.96%)	0 (0.0%)	0 (0.0%)
**Skin dryness**	64 (95.52%)	3 (4.48%)	0 (0.0%)	0 (0.0%)
**Skin and subcutaneous tissue disorders**	64 (95.52%)	3 (4.48%)	0 (0.0%)	0 (0.0%)

**Table 4:** Temperature Data Summary. A detailed summary of the temperature measurements recorded during HT sessions for both treatment groups is provided.

SCRT					
T10	T50	T90	Tmin	Tmax	Tavg
41.56 °C	40.31 °C	39.74 °C	38.60 °C	41.56 °C	40.31 °C
**LCRT**					
**T10**	**T50**	**T90**	**Tmin**	**Tmax**	**Tavg**
41.66 °C	40.32 °C	39.12 °C	38.52 °C	41.66 °C	40.05 °C

**Table 5:** Timing and duration of HT sessions. This table presents the time interval between the end of RT (beam-off) and the start of the HT session, as well as the duration of each HT session in minutes for both treatment groups (SCRT and LCRT).

Parameter	Group	Mean (Minutes)	Median (Minutes)	Range (Minutes)
**Interval time (beam-off to HT)**	**SCRT**	43.97	42	26–71
	**LCRT**	43.7	41	24–87
**HT session duration**	**SCRT**	54	60	4–61
	**LCRT**	50.9	60	2–62

## Data Availability

The data presented in this study are available on reasonable request from the corresponding author. The data are not publicly available due to privacy and ethical restrictions.

## Supplementary Materials

The following supporting information can be downloaded at: https://www.mdpi.com/article/10.3390/cancers17213529/s1, Table S1. Comparison of acute toxicities between patients treated with and without hyperthermia (HT).
