# Supplementary material for: Neoadjuvant Radiochemotherapy Combined with Locoregional Hyperthermia in Locally Advanced Rectal Cancer: Feasibility and Tolerance of Short-Course Versus Long-Course Radiotherapy Schedules
Source: Cancers (Basel). 2025 Oct 31;17(21):3529. doi: 10.3390/cancers17213529 (PMC12609664; doi:10.3390/cancers17213529)
Supplement: Supplementary file 1 [file cancers-17-03529-s001.zip › cancers-3926194-supplementary.pdf]

**Supplementary Table S1. Comparison of acute toxicities between patients treated with and without hyperthermia (HT).**
Data are presented as the number of patients (n) and percentage (%). Toxicity was graded according to CTCAE v4.03. No statistically significant differences were observed between groups.

| Toxicity (CTCAE v4.03) | Without HT (n = 53) | With HT (n = 67) | p-value |
|---|---|---|---|
| Anal pain | G1–2: 17.0% ≥G3: 1.9% | G1–2: 20.3% ≥G3: 0% | 0.72 |
| Anal ulcer | 0% | 0% | — |
| Constipation | G1–2: 20.8% ≥G3: 0% | G1–2: 25.4% ≥G3: 0% | 0.15 |
| Diarrhea | G1–2: 52.8% ≥G3: 3.8% | G1–2: 47.9% ≥G3: 3.4% | 0.51 |
| Fecal incontinence | 11.9% | 10.2% | 0.64 |
| Flatulence | 20.8% | 6.8% | 0.08 |
| Hemorrhoids | G1–2: 41.5% ≥G3: 0% | G1–2: 28.8% ≥G3: 6.8% | 0.12 |
| Nausea | G1–2: 30.2% ≥G3: 3.8% | G1–2: 17.0% ≥G3: 0% | 0.13 |
| Proctitis | G1–2: 22.7% ≥G3: 5.7% | G1–2: 24.4% ≥G3: 1.7% | 0.67 |
| Rectal fissure | 0% | 0% | — |
| Rectal fistula | 0% | 0% | — |
| Rectal bleeding | 1.9% | 5.1% | 0.36 |
| Rectal mucositis | G1–2: 45.3% ≥G3: 1.9% | G1–2: 35.8% ≥G3: 4.5% | 0.19 |
| Rectal obstruction | 0% | 0% | — |
| Rectal pain | G1–2: 47.2% ≥G3: 5.7% | G1–2: 47.4% ≥G3: 0% | 0.32 |
| Vomiting | G1–2: 7.5% ≥G3: 3.8% | G1–2: 1.7% ≥G3: 1.7% | 0.11 |
| Radiodermatitis | G1–2: 44.3% ≥G3: 7.5% | G1–2: 49.1% ≥G3: 6.8% | 0.64 |
| Myalgia | 0% | 1.7% | 0.34 |
| Anxiety | 5.7% | 6.8% | 0.84 |
| Dysuria | 24.5% | 16.9% | 0.32 |
| Hematuria | 0% | 1.7% | 0.34 |
| Urinary frequency | G1–2: 9.5% | 0% | 0.054 |
| Urinary incontinence | G1–3: 1.9% | G1–3: 1.7% | 0.37 |
| Urinary tract pain | 0% | 1.7% | 0.34 |
| Urinary urgency | G1–2: 3.8% | G1–2: 10.2% | 0.39 |
| Skin dryness | 0% | 5.1% | 0.096 |
| Skin/subcutaneous disorders | G1–2: 9.4% | G1–2: 5.1% | 0.27 |

**Note:** The comparison includes patients treated before, during, and after the implementation of deep regional hyperthermia within the institutional registry of the Radiation Oncology Department. Statistical tests (Chi-square or Fisher's exact test) showed no significant differences for any toxicity domain.
